# Translocation of Inhaled Ultrafine Manganese Oxide Particles to the Central Nervous System

**DOI:** 10.1289/ehp.9030

**Published:** 2006-04-20

**Authors:** Alison Elder, Robert Gelein, Vanessa Silva, Tessa Feikert, Lisa Opanashuk, Janet Carter, Russell Potter, Andrew Maynard, Yasuo Ito, Jacob Finkelstein, Günter Oberdörster

**Affiliations:** 1 Department of Environmental Medicine, University of Rochester, Rochester, New York, USA; 2 Procter and Gamble Co., Cincinnati, Ohio, USA; 3 Owens Corning, Granville, Ohio, USA; 4 Woodrow Wilson International Center for Scholars, Washington, DC, USA; 5 Department of Physics, Northern Illinois University, DeKalb, Illinois, USA; 6 Department of Pediatrics, University of Rochester, Rochester, New York, USA

**Keywords:** brain, central nervous system, CNS, inhalation, intranasal instillation, manganese, metals, nose, olfactory bulb, respiratory tract

## Abstract

**Background:**

Studies in monkeys with intranasally instilled gold ultrafine particles (UFPs; < 100 nm) and in rats with inhaled carbon UFPs suggested that solid UFPs deposited in the nose travel along the olfactory nerve to the olfactory bulb.

**Methods:**

To determine if olfactory translocation occurs for other solid metal UFPs and assess potential health effects, we exposed groups of rats to manganese (Mn) oxide UFPs (30 nm; ~ 500 μg/m^3^) with either both nostrils patent or the right nostril occluded. We analyzed Mn in lung, liver, olfactory bulb, and other brain regions, and we performed gene and protein analyses.

**Results:**

After 12 days of exposure with both nostrils patent, Mn concentrations in the olfactory bulb increased 3.5-fold, whereas lung Mn concentrations doubled; there were also increases in striatum, frontal cortex, and cerebellum. Lung lavage analysis showed no indications of lung inflammation, whereas increases in olfactory bulb tumor necrosis factor-α mRNA (~ 8-fold) and protein (~ 30-fold) were found after 11 days of exposure and, to a lesser degree, in other brain regions with increased Mn levels. Macrophage inflammatory protein-2, glial fibrillary acidic protein, and neuronal cell adhesion molecule mRNA were also increased in olfactory bulb. With the right nostril occluded for a 2-day exposure, Mn accumulated only in the left olfactory bulb. Solubilization of the Mn oxide UFPs was < 1.5% per day.

**Conclusions:**

We conclude that the olfactory neuronal pathway is efficient for translocating inhaled Mn oxide as solid UFPs to the central nervous system and that this can result in inflammatory changes. We suggest that despite differences between human and rodent olfactory systems, this pathway is relevant in humans.

An important step in assessing the toxicology of particles is to determine their fate after inhalation. Of particular interest to us are airborne ultrafine particles (UFPs; < 100 nm), which are abundant in ambient urban air and are of the same size as engineered nanoparticles. Translocation to extrapulmonary sites after respiratory tract deposition represents an important mechanism for these particles to cause direct effects in secondary target organs (Oberdörster et al. 2005). The extent to which this process occurs depends on several factors including particle solubility, particle or aggregate size, the site of deposition, and the integrity of the epithelial lining. UFPs deposit efficiently in all regions of the respiratory tract, depending on their size; specifically, as particle size decreases toward the smallest UFPs, nasopharyngeal deposition increases (International Committee on Radiological Protection 1994).

Studies in rats have shown translocation of soluble manganese compounds from the nose along olfactory neuronal pathways to the olfactory bulb (Dorman et al. 2004; Henriksson and Tjälve 2000; Tjälve et al. 1996; Tjälve and Henriksson 1999) after inhalation or intranasal instillation exposures. Likewise, the few studies that have examined the fate of UFPs deposited on the nasal mucosa identified translocation along the neuronal olfactory route as a pathway to the olfactory bulb of the central nervous system (CNS). These include early studies in non-human primates, which demonstrated the translocation of solid nanosized particles (30 nm poliovirus; 50 nm silver-coated gold colloids) along the axons of olfactory nerves into the olfactory bulb (Bodian and Howe 1941a, 1941b; DeLorenzo 1970). We have also shown that inhaled elemental carbon particles (^13^C; 35 nm, count median diameter) accumulate in rat olfactory bulb after whole-body inhalation (Oberdörster et al. 2004). Regarding penetration into deeper brain regions, Tjälve et al. (1995) demonstrated that soluble ionic Mn instilled into the olfactory chamber of pike has the ability to pass synaptic junctions and migrate from the olfactory tract to more distal regions, including the hypothalamus. Dorman et al. (2004) found Mn in the striatum and cerebellum of rats after subchronic inhalation exposure to a soluble Mn salt (sulfate); however, this was attributed to uptake from the blood. Thus, contributions to brain Mn levels from the blood need to be considered and may also be an issue for inhaled solid UFPs.

The effects of translocated particles in the brain are also important to determine. For example, preliminary information has emerged from populations of welders that some of them may develop parkinsonism 17 years earlier than the general population (Racette et al. 2001). Welding produces high amounts of fumes containing Mn UFPs (Zimmer et al. 2002). Several recent epidemiologic studies describe occupational exposure ranges of approximately 0.01–5 mg/m^3^ Mn in fumes from various welding processes and materials (Korczynski 2000; Li et al. 2004; Sinczuk-Walczak et al. 2001). Conflicting data emerge from animal studies, however, regarding effects of inhaled Mn compounds in the brain. Henriksson and Tjälve (2000) reported changes in glial fibrillary acidic protein (GFAP) and S-100b, markers of astrocyte activation, in several brain regions from rats exposed intranasally to Mn chloride. However, Dorman et al. (2004) did not find any evidence of changes in GFAP levels in the brain after exposure to Mn sulfate or phosphate. Potential contributing factors to the lack of concurrence in results include differences in the solubilities of the Mn salts used, the doses, and the contribution of olfactory epithelial damage.

In the present study, we sought to address the hypothesis that a major translocation route for inhaled poorly soluble Mn oxide UFPs from deposits in the nose is to the olfactory bulb in the CNS. We characterized the size, oxidation state, and *in vitro* solubility of gas-phase–generated Mn oxide particles and also compared the translocation kinetics to the olfactory bulb of Mn oxide and MnCl_2_ that were applied to the nasal epithelium of rats via instillation. We then measured the accumulation of Mn in lung, liver, and olfactory bulb after repeated inhalation exposures with both nares patent or with one naris occluded. We show that Mn oxide UFPs are translocated to and retained in the olfactory bulb (ipsilateral to the patent naris only) and present evidence of exposure-induced effects in that region of the brain. These studies demonstrate the importance of UFPs size and of solubility in olfactory translocation processes.

## Materials and Methods

### Animals

Specific pathogen-free male Fischer 344 rats (200–250 g body weight, 3 months of age) were obtained from Harlan (Indianapolis, IN) and housed in filter-top plastic cages in a facility accredited by the International Association for Assessment and Accreditation of Laboratory Animal Care; animals had free access to Purina rodent chow (5001; Purina Mills, LLC, St. Louis, MO) and water. The background concentration of particles in this facility is extremely low (< 50 particles/cm^3^). All animals were allowed to acclimate for at least 1 week before use in experimental protocols, which were approved by the University Committee on Animal Resources (University of Rochester). Animals were treated humanely and with regard to the alleviation of suffering.

### Generation of Mn oxide UFPs

We generated Mn oxide UFPs (~ 500 μg/m^3^; 18 × 10^6^ particles/cm^3^) via electric arc discharge (Palas GmbH, Karlsruhe, Germany) in an argon-filled chamber between two opposing Mn rods (purity, 99.95%; Electronic Space Products International, Ashland, OR). Oxygen was introduced into the generator (20 mL/min) to ensure the formation of the metal oxide. Particle electrostatic charge was brought to Boltzman equilibrium (^210^Po source). Available instrumentation monitored particle mass and number concentrations continuously as well as aerosol size distribution at regular intervals [tapered element oscillating microbalance (Rupprecht and Patashnik, Albany, NY); condensation particle counter, model 3022A, and scanning mobility particle sizer, model 3080 (TSI Inc., St. Paul, MN)].

Gas-phase–generated Mn oxide UFPs were collected for electron microscopic analyses immediately after generation using a point-to-plane electrostatic precipitator for transmission electron microscopy (TEM) samples (In-Tox Products, Moriarty, NM). For scanning electron microscopy (SEM) analysis, the particles were suspended in methanol, one drop placed on an SEM stub and desiccated for 24 hr. Electron micrographs (Figure 1S in Supplemental Material available online at http://www.ehponline.org/docs/2006/9030/suppl.pdf) showed that the aerosol was composed of agglomerates with an equivalent sphere diameter of approximately 30 nm. Agreement between scanning mobility particle sizer–derived and TEM-derived size distributions was very good. Primary particles varied between 3 and 8 nm in diameter, depending on the generation conditions.

### Exposure of rats to Mn oxide UFPs

For whole-body inhalation exposures, rats were placed in compartmentalized, horizontal flow chambers (31-L Lucite tank; airflow = 30 L/min). In some exposures, the right naris was occluded according to methods outlined by Brenneman et al. (2000). Briefly, a 3-mm piece of polyethylene tubing [0.024 in. outer diameter (OD)] was inserted into an 8-mm piece of Silastic tubing (0.065 in. OD), the ends of which were sealed to form smooth, round edges. One day before exposure, rats were lightly anesthetized with Halothane (Cardinal Health Pharmaceutical Distribution, Syracuse, NY), and the plug was inserted into the right naris; a small amount of Duro Quick Gel (Ethicon, Inc., Somerville, NJ) ensured that the plug remained in place. Inhalation exposures were for 6 hr/day, 5 days/week for up to 12 days with both nares open (12 days total for tissue Mn determinations; 11 days total for gene and protein array analyses). With one naris occluded, the exposure was for 2 days.

For intranasal instillation exposures, the particles were collected on a filter and resuspended in sterile pyrogen-free saline with sonication. Because nasally instilled material is readily aspirated into the lower respiratory tract in rodents, thus making olfactory mucosa exposure less than optimal, we developed a simple method to maximize the dose to the olfactory mucosa. Rats were lightly anesthetized with Halothane (3%). The trachea was visualized with a pediatric otoscope and cannulated transorally with the plastic sheath of a 14-gauge catheter. The plane of anesthesia was maintained with Halothane (1.5%). Breathing through the tracheal cannula ensured that aspiration of the nasal instillate into the lung did not occur. A 30-gauge needle covered with polyethylene tubing was attached to a 1-mL syringe and the tubing was inserted into the left naris (5 mm), after which 5–7 μg (in 30 μL) of the suspended particles was slowly injected with the rats in a supine position. The rats were kept supine for 5 min, after which the Halothane was turned off and the tracheal cannula was removed once the animal regained consciousness. These methods were used for both Mn oxide and MnCl_2_ exposures.

### Characterization of the oxidation state and oxide form of Mn oxide UFPs

We investigated the oxidation state of sampled Mn oxide particles using electron energy loss spectroscopy (EELS; GIF 2000 system; Gatan Inc., Pleasanton, CA) and an analytical TEM/STEM (Tecnai F20ST; FEI Co., Hillsboro, OR) operating in scanning transmission electron microscopy (STEM) mode. Mn L-electron and oxygen K-electron energy loss edges were recorded from discrete primary particles and clusters of particles. We estimated elemental stoichiometry through comparison of the integrated area under energy loss edges after background subtraction, using Digital Micrograph (Gatan Inc.). Mn oxidation state was further characterized by comparing acquired spectra with published Mn L-electron X-ray absorption spectra for Mn oxide (MnO), Mn tetroxide (Mn_3_O_4_), manganese sesquioxide (Mn_2_O_3_), and Mn dioxide (MnO _2_ ) (Gilbert et al. 2003). Further information on the calculation of the relative percentages of each state is given in Supplemental Material available online (http://www.ehponline.org/docs/2006/9030/suppl.pdf).

### Dissolution rate of Mn oxide UFPs

We employed two methods to determine the dissolution rate of the gas-phase–generated Mn oxide UFPs in solution: ultrafiltration and dialysis. For ultrafiltration, samples (0.5 mg) were dispersed in Mn-free physiologic saline (1 mL, pH 7.4) using an ultrasonic bath. Sample suspensions were injected into the inlet flow line of the ultrafiltration sample cell (Molecular/Por stirred cell, stirring system removed; Spectrum Laboratories Inc., Rancho Dominguez, CA) fitted with a 1,000-molecular-weight cutoff membrane (Molecular/Por cellulose ester ultrafiltration membrane; Spectrum Laboratories Inc.). The sample was washed into the cell with an additional 2 mL saline solution. For dialysis, the sample suspensions were injected into the upper chamber of a dialysis cell (Spectra/Por MacroDialyzer, Spectrum Laboratories Inc.) fitted with a 1,000-molecular-weight cutoff membrane (Molecular/Por cellulose ester asymmetric membrane; Spectrum Laboratories Inc.). The sample cells were then swirled to evenly disperse the suspensions; after several hours, the suspensions settled as an even layer on the membrane filters. Inlet ports were connected to a peristaltic pump for optimal flow; the outlet ports were connected to a waste bottle. Outlet lines were periodically connected to sample tubes, and 50 mL of the filtrate or dialysate was collected and analyzed for Mn by inductively coupled plasma optical emission spectroscopy (ICP-OES). For measurements of the dissolution rate above room temperature, the entire sample cell was immersed in a water bath at the desired temperature.

### Cellular and biochemical parameters in bronchoalveolar lavage fluid

Animals were euthanized with an overdose of sodium pentobarbital (intraperitoneal, 50 mg/100 g body weight) followed by exsanguination. As described in detail elsewhere (Elder et al. 2000), the lungs, trachea, and heart were removed *en bloc*, and the right lungs were lavaged after weighing with a fixed volume of sterile, pyrogen-free 0.9% saline (five times, each with 5 mL), separating the first two lavages for protein and enzymatic analyses. Cells were pelleted by centrifugation (400 × *g*) for 10 min. The cells were pooled from all lavage fractions for viability determination (trypan blue exclusion), enumeration, and differential analysis (Diff-Quik; Baxter Scientific, Edison, NY). Total protein concentration and lactate dehydrogenase and β-glucuronidase activities were measured using commercially available kits (Pierce Chemical Co., Rockford, IL; Sigma, St. Louis, MO).

### Analyses of metal content in tissue samples

At the time of sacrifice, we removed the skin and fur from the rats with a dedicated set of instruments, and the carcass was thoroughly washed before the removal of tissues; the carcasses were then moved to a separate room for excision of organs to be analyzed for metals content with different sets of instruments. The left lungs, 1 g of liver tissue, and several brain regions (olfactory bulbs, striatum, trigeminal ganglions, midbrain, frontal cortex, cortex, cerebellum) were removed to measure their Mn and iron content. In the intranasal instillation studies, right and left olfactory bulbs and mucosa (including turbinates and cribriform plate) were removed. We did not perfuse the tissues because preliminary tests showed that perfusion did not affect Mn levels. The tissues were placed directly into Teflon digestion vials, weighed, and wet ashed with ultrapure 70% nitric acid (Baseline, SeaStar Chemicals Inc., Sidney, British Columbia, Canada). After ashing, the tissue residue was resuspended in HNO_3_ and the concentration was adjusted to 2% with 18 M Ω deionized water before graphite furnace atomic absorption spectroscopy analysis. Quantitation was achieved through comparison to reference standards (Standard Reference Material 1577b from bovine liver; National Institute of Standards and Technology, Gaithersburg, MD).

### RNA preparation for array and blot analysis

Tissue samples were prepared as previously described (Carter and Driscoll 2001) using the standard RNAzol protocol for array and blot analysis. RNA was amplified using the primer-specific Smart Probe Amplification Kit (Clontech, Palo Alto, CA) designed specifically for the Atlas array systems. Briefly, 50 ng of total RNA was amplified using specific primer sets for reverse-transcriptase polymerase chain reaction (PCR) amplification to full length double-stranded cDNA, which ensures amplification of representative original gene population. Double-stranded cDNA generated from the PCR amplification step was pooled separately from control and treated groups, respectively, and cDNAs were prepared with standard reagents and the Rat 1.2 and Rat 1.2 II Array kits (Clontech) following the manufacturer’s instructions. Labeled cDNA was generated during the first strand synthesis supplemented with [α-32P]-deoxyadenosine 5′-triphosphate (3,000 Ci/mmol, 10 μCi/μL) using Moloney Murine Leukemia Virus (MMLV) reverse transcriptase (Clontech) and purified by column chromatography. Details of array membrane hybridization and array analyses are given in Supplemental Material available online (http://www.ehponline.org/docs/2006/9030/suppl.pdf)

### Protein array analysis

Protein was extracted from lung and brain samples using the BD Clontech Protein Extraction and Labeling Kit (Clontech). Briefly, 1 μg of protein from each sample was pooled and used to probe the RayBio Rat Cytokine Array I according to the manufacturer’s protocol (RayBioTech, Norcross, CA) (Lin et al. 2003). After 20 min exposure, the membranes were scanned using a phosphorimager (BioRad Molecular Imager Fx; Bio-Rad Laboratories, Hercules, CA) and analyzed for relative intensity of expression. Data are expressed as fold change differences from the unexposed controls.

### Statistical analyses

We analyzed results for statistical differences by one-way analysis of variance with appropriate data transforms using SigmaStat (Systat Software Inc., Point Richmond, CA). Data were appropriately transformed if an analysis of residuals suggested deviations from the assumptions of normality and equal variance. Differences between groups were further analyzed using Tukey multiple comparisons. Such comparisons were considered statistically significant when *p* ≤ 0.05.

## Results

### Tissue distribution of Mn and effects in the lungs and brain after repeated inhalation exposure to ultrafine Mn oxide aerosols

**Figure 1:**
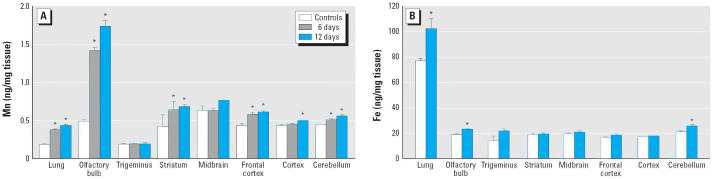
Mn and Fe contents in lung and brain tissues after 6 and 12 days of inhalation exposure to ultrafine Mn oxide aerosols. (*A*) Mn content in lung and brain tissues in controls (*n* = 5) and after 6 (*n* = 3) and 12 (*n* = 3) days of exposure. (*B*) Fe content in lung and brain tissues in controls (*n* = 5) and after 12 days of exposure (*n* = 3). Values are mean ± SE. *p < 0.05 versus filtered air-exposed controls.

We exposed rats to either filtered air or to ultra-fine Mn oxide aerosols (465 ± 94 μg/m^3^) for 6 hr/day for 12 days. There were no indications in terms of either cellular or biochemical parameters to indicate that active lung inflammation occurred as a result of exposure (Table 1); the only statistically significant changes found were decreases in lavage fluid total cell number after 6 days of exposure and β-glucuronidase activity after 12 days of exposure. The Mn content in lung tissue increased progressively and significantly after 6 and 12 days of ultrafine Mn oxide aerosol exposure (Figure 1A), representing about a doubling of the tissue Mn content. Inhalation of Mn oxide aerosols for 12 days also resulted in significant increases (~ 3.5-fold) in olfactory bulb Mn content. Significant increases in Mn content were also found in the striatum, frontal cortex, and cerebellum after 6 and 12 days of exposure and in the cortex after 12 days of exposure (Figure 1A), although the magnitude of these changes was much lower than what was observed in olfactory bulb. The increase in liver Mn content was smaller than the increase in lung tissue (controls, 2.78 ± 0.04 ng Mn/mg tissue; 6 days, 2.61 ± 1.61 ng Mn/mg tissue; 12 days, 2.91 ± 0.09 ng Mn/mg tissue). The tissue concentration of Fe also increased slightly, but significantly, after exposure in lung, olfactory bulb, and cerebellum (Figure 1B).

**Figure 2:**
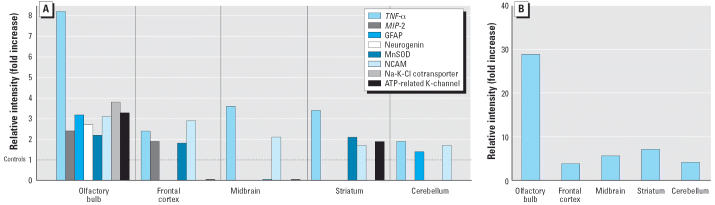
Gene and protein expression changes, by brain region, from pooled samples after 11 days of inhalation exposure to ultrafine Mn oxide aerosols. (*A*) Gene expression changes represented as relative intensities (fold increase over normalized control, dashed line). (*B*) TNF-α protein expression changes (relative intensities, fold increase over normalized control). Abbreviations: MIP-2, macrophage inflammatory protein-2; MnSOD, manganese superoxide dismutase; NCAM, neuronal cell adhesion molecule.

Gene microarray analyses were performed on tissue samples obtained from various brain regions after a total of 11 days of ultrafine Mn oxide aerosol exposure (498 ± 69 μg/m^3^). Figure 2A shows the relative increases in expression for selected genes. Tumor necrosis factor-α (*TNF*-α) gene expression was increased in olfactory bulb, frontal cortex, midbrain, and striatum. Protein expression of TNF-α correlated with gene expression increases (Figure 2B). Several other genes involved in inflammation (e.g., macrophage inflammatory protein-2) and stress responses (e.g., GFAP) also showed 2-fold or greater increases in expression over controls in the olfactory bulb. Although most of the changes occurred in olfactory bulb tissue, neuronal cell adhesion molecule was also increased in the frontal cortex and midbrain (Figure 2A). Several other genes exhibited increases in expression that were slightly less than 2-fold higher compared with controls.

### Tissue distribution of Mn after inhalation of ultrafine Mn oxide aerosols with one naris occluded

The data presented above show that Mn accumulation was the highest in the olfactory bulb and lower in other brain regions, consistent with olfactory translocation after repeated inhalation exposures; however, the Mn in those tissues could have arisen from bloodborne Mn. In a separate set of experiments, rats were exposed with the right nares occluded for 6 hr on each of 2 consecutive days. Three animals each were killed for tissue Mn analyses immediately after the first exposure and 18 hr after the first and second exposures. Control animals with both nares open were killed 18 hr after a 6 hr exposure; there were also unexposed controls (0 hr). With both nares patent, significant increases in lung Mn content were found 18 hr after a 6-hr ultrafine Mn oxide aerosol exposure (Figure 2S in Supplemental Material available online at http://www.ehponline.org/docs/2006/9030/suppl.pdf), as was expected from the repeated inhalation studies; liver Mn content also increased. When the right naris was occluded during exposure, lung tissue Mn content increased 11-fold to 1.9 ± 0.05 ng/mg wet tissue immediately after the 6-hr exposure; liver Mn content increased from 2.5 ± 0.01 to 2.9 ± 0.08 ng/mg tissue. After 18 hr of recovery, significant amounts of Mn remained in the lung (21% of the amount deposited at the end of exposure), but most of the deposited material had been cleared, and no increase was observed in liver tissue. Two consecutive days of exposure (6 hr each) did not significantly alter the retention kinetics of the ultrafine Mn oxide aerosols deposited in the lung (Figure 2S in Supplemental Material available online at http://www.ehponline.org/docs/2006/9030/suppl.pdf). Thus, clearance of Mn from the lung under these exposure conditions is rapid.

**Figure 3:**
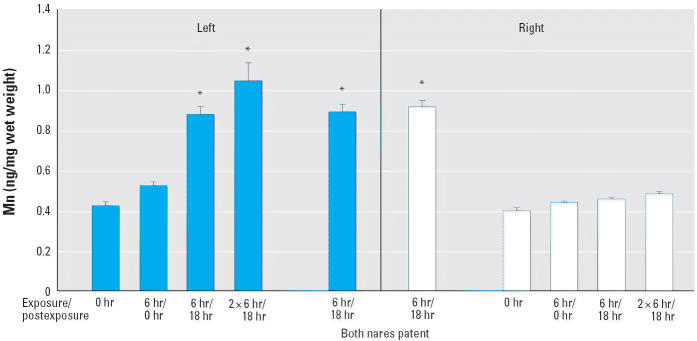
Accumulation of Mn in right and left olfactory bulb after inhalation exposure to ultrafine Mn oxide aerosols with the right naris occluded. Exposure duration and postexposure time are shown on the *x*-axis. Tissues were obtained from the same rats as in Figure 2S in Supplemental Material available online (http://www.ehponline.org/docs/2006/9030/suppl.pdf). Values are mean ± SE. *p < 0.05 versus 0 hr.

Mn also accumulated in the left and right olfactory bulbs when both nares were patent (Figure 3). However, when the right naris was occluded, Mn accumulated only in the olfactory bulb on the patent (left) side. Furthermore, Mn accumulation increased in left olfactory bulb tissue, unlike the lung, with time postexposure and exposure duration. A small, but insignificant, amount of Mn also accumulated in the right olfactory bulb after two consecutive 6-hr exposures (1.2-fold increase), potentially from bloodborne Mn due to dissolution of the Mn oxide in alveolar macrophages (Lundborg and Camner 1984; Lundborg et al. 1985) or due to transport between the nares via a small perforation in the rat nasal septum, as has been previously described (Kelemen and Sargent 1946).

### Mn oxide UFP oxidation state and solubility

**Figure 4:**
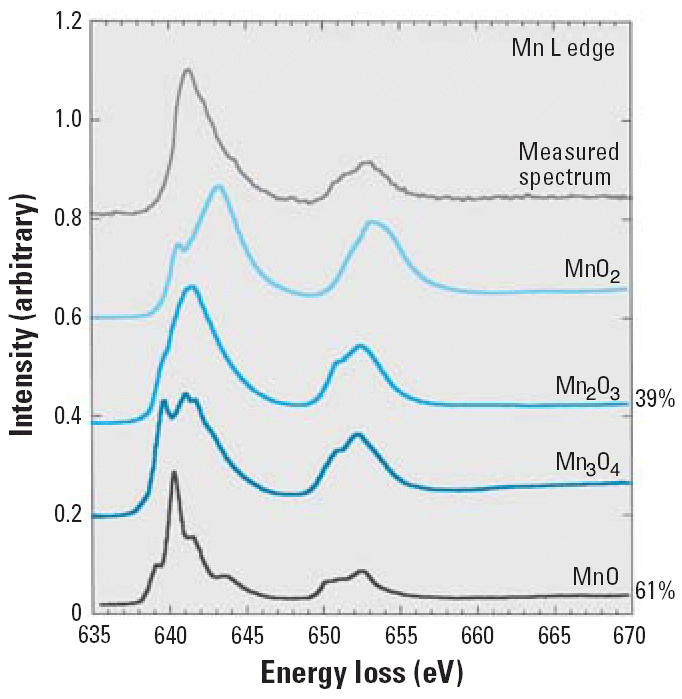
Approximate identification of component oxides by comparing measured EELS edge structure of gas-phase–generated Mn oxide to reference oxide spectra.

EELS analysis of primary particles and agglomerates indicated a mean stoichiometry close to MnO. Comparison of Mn L-electron edge structure with published spectra for different oxidation states indicated that the particles were composed of MnO [61%, Mn(II)] and Mn_2_O_3_ [39%, Mn(III)] (Figure 4). At room temperature, both the ultrafiltration and dialysis experiments showed that the Mn oxide UFPs dissolved at a rate of 1–1.5% per day (i.e., Mn detected in outflow via ICP-OES) at a neutral pH similar to the nasal mucosal milieu. Subsequent dialysis experiments also showed that temperature, solution flow rate, and time (up to 10 days) did not affect the dissolution rate. However, acidification to pH 4.5, similar to the phagolysosomal conditions of alveolar macrophages (Lundborg et al. 1985), resulted in rapid dissolution. This soluble fraction most likely contributed to bloodborne Mn that was then distributed throughout the body.

### Olfactory bulb uptake of Mn after intra-nasal instillation of solid ultrafine Mn oxide or a soluble Mn salt

**Figure 5:**
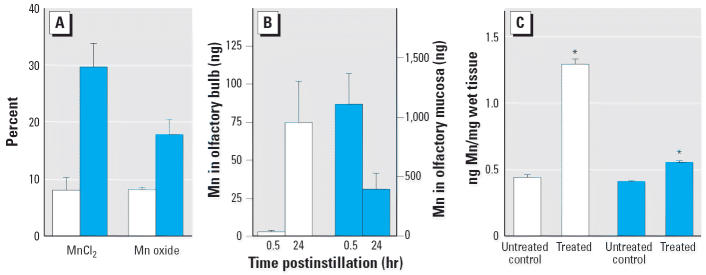
Translocation of instilled Mn to olfactory bulb in separate experiments. (*A*) Percentage of instilled Mn retained in olfactory bulb (shaded bars) and retained in olfactory mucosa (solid bars) 24 hr after instillation of either MnCl_2_ or Mn oxide into left naris, *n* = 3/group. (*B*) Background corrected amount of Mn in olfactory bulb (shaded bars) and on olfactory mucosa (solid bars) at 30 min and 24 hr after instillation of Mn oxide into the left naris, *n* = 3/group. (*C*) Amount of Mn in left (shaded bars) and right (solid bars) olfactory bulb tissue from untreated control rats (*n* = 5) and 24 hr after instillation of Mn oxide into the left naris (*n* = 6 rats). Values are means ± SE. *p < 0.05 versus untreated controls.

The issue of whether or not the solubilization of ultrafine Mn oxide was a prerequisite for its translocation along the olfactory nerve was still outstanding after the experiments described above. Given the low solubilization rate (1–1.5% per day) of ultrafine Mn oxide at neutral pH, one would predict that only this small fraction of Mn oxide deposited on the olfactory mucosa would be translocated to the olfactory bulbs in its soluble form. In order to test this, we instilled 30 μL of soluble MnCl_2_ (5.3 μg Mn) or saline-suspended Mn oxide UFPs (6.7 μg Mn) into the left naris of anesthetized rats. Despite the fact that only 1.5% of the Mn in the oxide form was soluble at a maximum, similar Mn burdens (given as a percentage of the instilled Mn) were found in the left olfactory bulb tissue 24 hr after intranasal instillation of the chloride (8.2 ± 3.6%) or the oxide (8.2 ± 0.7%). Furthermore, less Mn from the oxide was found on the cribriform plate area and the turbinates (representative of the olfactory mucosa), indicating greater retention of the MnCl_2_ by those tissues (Figure 5A). In another group of rats, olfactory bulb uptake of intranasally instilled Mn oxide occurred rapidly (within 30 min) after exposure, although to a very small degree (0.2% of amount found on olfactory mucosa at 30 min); however, this had increased to 6.8% after 24 hr (Figure 5B). In another experiment, Mn oxide UFPs that were instilled into the left naris translocated primarily to the left olfactory bulb, although a small amount also appeared in the right olfactory bulb, possibly due to some exposure of the right side of the nose via the nasal septal window (Kelemen and Sargent 1946) (Figure 5C). These data indicate that the appearance of Mn in olfactory tissues cannot be due to soluble Mn but, rather, that solid UFP transport occurs efficiently.

## Discussion

Traditionally, the respiratory tract is considered a target organ for effects of inhaled solid particles. However, more recent evidence from epidemiologic, controlled clinical and animal studies with ambient particulate air pollutants shows that extrapulmonary organs are also affected (U.S. EPA 2004). Specifically, it has been hypothesized that inhaled UFPs accumulate and cause effects in extrapulmonary organs, such as the cardiovascular system and CNS (Donaldson and Stone 2003; Oberdörster et al. 2005; Oberdörster and Utell 2002) because of their propensity to translocate across epithelial barriers. Indeed, in our present study we demonstrate that 6- to 12-day inhalation exposure of rats for to solid Mn oxide UFPs resulted in significant increases of Mn in several brain regions, most notably the olfactory bulb. The fact that occlusion of the right naris during inhalation for 2 days of the nanosized Mn oxide led to accumulation of Mn only in the left olfactory bulb confirmed that translocation from nasal deposits along the olfactory nerve accounts for this increase.

The olfactory translocation route has been well demonstrated for soluble Mn compounds (Dorman et al. 2004; Tjälve and Henriksson 1999), and it has been suggested that solubility is an important determinant of the efficiency of this process (Dorman et al. 2001). Dorman et al. (2004) measured the translocation of poorly soluble Mn phosphate into olfactory bulb, cerebellum, and striatum. In that study, only the olfactory bulb, and not the more distal structures, demonstrated a small increase in Mn content. One important difference to our study, however, is that the phosphate particles had a mass median aerodynamic diameter of 1.6 μm, whereas the count median diameter of the Mn oxide particles used here was 31 nm. The axons of olfactory neurons narrow to a diameter of approximately 200 nm and become tightly packed where they pass through the cribriform plate pores (DeLorenzo 1957; Plattig 1989). Thus, in order for solid particles deposited on the olfactory mucosa to be transported through the cribriform plate, they should be < 200 nm in size. We suggest, therefore, that particle size plays a most important role and that dissolution is not a prerequisite for neuronal uptake and translocation of solid UFPs in the absence of mucosal injury. Indeed, this suggestion is based on earlier studies in nonhuman primates by Bodian and Howe (1941a, 1941b) and by DeLorenzo (1970) with intranasally instilled 30 nm poliovirus and 50 nm silver-coated gold particles, respectively, and on our recent study in rats with inhaled carbon-13 particles (median size, ~ 30 nm; Oberdörster et al. 2004) showing translocation along olfactory neuronal pathways.

The Mn oxide UFPs used in the present study dissolved at a rate of about 1.5% over 24 hr in physiologic saline at neutral pH. However, greater solubilization of Mn oxide has been demonstrated at approximately pH 4.5, as would be encountered in the phagolysosome of alveolar macrophages (Lundborg and Camner 1984; Lundborg et al. 1985). Given that the nasal mucosa has a pH close to neutral (Washington et al. 2000) and free macrophages are not normally present on the nasal epithelial surface (Harkema J, personal communication), rapid solubilization of the intranasally instilled Mn oxide UFPs in our study is unlikely. We conclude, therefore, that the increase in olfactory bulb Mn in our study is due to neuronal uptake and translocation of the solid Mn oxide UFPs. Support for this suggestion comes also from the results of our comparison of intranasally instilled Mn oxide to soluble MnCl_2_. If solubilization of the Mn oxide UFPs were a prerequisite for neuronal translocation of Mn, one would expect that significantly less Mn would accumulate in the olfactory bulb for instilled Mn oxide compared with MnCl_2_ because of the low solubilization rate of the oxide. Instead, it was the same for both compounds. In addition, one would expect more of the Mn oxide than the chloride to be retained on the olfactory mucosa; however, the opposite was found (Figure 5A). Finally, the accumulation kinetics are consistent with a rapid translocation velocity of solid particles in axons (up to 6 mm/hr; Adams and Bray 1983).

Another issue to consider is that Mn oxide UFPs that deposited in the alveolar compartment of the rat respiratory tract in our study may have undergone dissolution in alveolar macrophages (Lundborg and Camner 1984; Lundborg et al. 1985) followed by diffusion of soluble ionic Mn into the blood circulation. In addition, Mn oxide UFPs may translocate across the alveolar–capillary barrier and the blood–brain barrier in regions where it is discontinuous (e.g., choroid plexus, ventricles, brain stem, hypothalamus). Dissolution and absorption of Mn oxide particles deposited in the respiratory tract and cleared into the GI tract via mucociliary action could also contribute to bloodborne Mn; however, this will only be a small fraction, < 5% of the ingested amount (Oberdörster and Cherian 1988). Although it is likely that bloodborne ionic Mn contributed to the increase in cerebellar Mn, the degree to which this may have contributed to Mn in other brain regions is not known. It is also possible that Mn crosses the blood–brain barrier via competition with Fe (Aschner et al. 2005); both Fe and Mn can complex with transferrin in the blood and thereby compete for uptake into the CNS capillary endothelium via the transferrin receptor (Malecki et al. 1999). In addition, Mn appears to facilitate the transport of Fe into the brain after chronic exposure (Zheng et al. 1999). Our finding of higher Fe levels in olfactory bulb and cerebellum after Mn oxide inhalation (Figure 1) may be related to competitive or facilitated transport, but this needs to be investigated in further studies (Fe was not present in the exposure atmosphere). Because the tissues were not perfused, some of the Fe could have come from the blood, and future studies should be done using perfused tissues.

Frontal cortex and striatal Mn increases in our study may have been due either to a contribution from bloodborne Mn or to neuronal translocation from olfactory bulb to more distal brain structures. Large molecules, approaching the size of nanoparticles (wheat germ agglutinin–horseradish peroxidase complex), were found to translocate from the nasal cavity to the olfactory bulb and more distal structures by crossing synapses (Shipley 1985), as was also observed with herpes virus (McLean et al. 1989). Thus, although ionic bloodborne Mn can contribute to increased Mn levels in some brain regions, translocation of solid Mn oxide UFPs from nasal deposits to CNS regions via the olfactory nerve should not be excluded as a possible mechanism.

Using a predictive particle deposition model for the rat (multiple path particle dosimetry model; Asgharian and Anjilvel et al. 1998), we estimated from our data that about 11.5% of the amount deposited on the olfactory mucosa (369 ng) was translocated to the olfactory bulb (42.5 ng). Model inputs included the Mn oxide particle size distribution and exposure concentration; the default breathing parameters for rats (tidal volume, 2.1 mL; respiratory rate, 102/min) and the rat-specific nasal parameters given in Table 1S (in Supplemental Material available online at http://www.ehponline.org/docs/2006/9030/suppl.pdf) were also considered. Olfactory bulb weights and Mn concentrations were those from experimental results found in Figure 3; the olfactory bulb Mn background level in unexposed rats was subtracted from the level in rats exposed to Mn oxide for 6 hr with both nares patent.

The results of this study raise a number of important questions, including extrapolation to humans, the significance for neurotoxicity, implications for other inhaled nanosized particles, and translocation from olfactory bulb to deeper brain structures. Human exposures to high concentrations of Mn oxide–containing UFPs occur under certain occupational settings, such as arc welding (Zimmer et al. 2002). Concentrations of UFPs containing Mn oxide can reach 10^7^ particles/cm^3^. The issue of the neurotoxicity of inhaled Mn-containing particles is important insofar as welding fumes contain 0.01–5 mg/m^3^ Mn, depending on the welding process and materials used (Korczynski 2000; Li et al. 2004; Sinczuk-Walczak et al. 2001), and preliminary results from a small cohort of welders show that they may develop Parkinson-like symptoms (Racette et al. 2001). Dorman et al. (2004) showed that the inhalation of soluble Mn sulfate aerosols did not lead to an increase in GFAP protein expression in olfactory bulb tissue; however, Henriksson and Tjälve (2000) performed three successive weekly intranasal instillations of MnCl_2_ and showed an increase in this protein, which is a marker of astrocyte reactivity, or response to injury. An important point, however, is that increased GFAP was noted by Henriksson and Tjälve (2000) only at high doses that also led to olfactory epithelial damage. In the present study, we found evidence for increased *GFAP* and *TNF-*α gene expression, among other things, in olfactory bulb tissue; although TNF-α message expression was elevated in deeper brain structures, this was not the case for GFAP (Figure 2A). TNF-α protein expression was also increased in those brain regions where its gene expression was increased (Figure 2B).

We did not perform histopathologic assessment of the respiratory or olfactory epithelium and thus do not know if the Mn oxide UFPs caused inflammation at the olfactory mucosa, which could have affected neuronal uptake and translocation. A significant nasal inflammatory response may be unlikely given that there was no inflammation in the lung after 12 days of exposure (Table 1). Even a 1-day exposure—which is far less likely to induce nasal inflammation—resulted in significant translocation to the olfactory bulb (Figure 3). Our observation of a decrease in lung lavage fluid β-glucuronidase activity after 12 days of exposure is similar to the findings of Bairati et al. (1997), who reported decreases in the activity of this enzyme in plasma from humans exposed occupationally to Mn and lead. These changes were used as a marker of heavy metal exposure. Because the physiologic and pathologic implications of these changes are unclear, histologic evaluations of lung and liver after chronic exposures in animals should be performed in future studies. Our results with MnCl_2_ suggest that it is retained to a greater extent than the oxide in olfactory mucosa (Figure 5B), thus possibly explaining the damage to those tissues by more soluble forms of Mn delivered at high doses.

The earlier studies of olfactory translocation of nanosized particles of different types (viruses, gold, carbon) together with the present study of ultrafine Mn oxide may imply that all nanosized particles deposited on the olfactory mucosa will translocate to the brain. However, uptake into sensory nerve endings and subsequent translocation is likely to depend not only on size, but also on many other particle characteristics, such as shape, chemistry, surface properties (area, porosity, charge, surface modifications), agglomeration state, solubility, and dose. Although there are no data regarding these parameters for sensory neurons, they affect endo- and transcytosis of nanosized materials (e.g., Kato et al. 2003; Kreuter 2004; Rejman et al. 2004). However, our findings may not be directly applied to nanoparticles in general until more data are available on mechanisms controlling neuronal uptake and translocation.

We conclude from our studies that the olfactory neuronal pathway represents a significant exposure route of CNS tissue to inhaled solid Mn oxide UFPs. In rats, which are obligatory nose breathers, translocation of inhaled nanosized particles along neurons seems to be a more efficient pathway to the CNS than via the blood circulation across the blood–brain barrier. Given that this neuronal translocation pathway was also demonstrated in nonhuman primates, it is likely to be operative in humans as well.

## Correction

In “Materials and Methods” in the original manuscript published online, the authors incorrectly stated that animals were housed in wire-bottom cages; this has been corrected here.

## Figures and Tables

**Table 1:** Summary of lavage data from 6- and 12-day ultrafine Mn oxide exposures in young male F-344 rats.

		Mn exposure	
	Untreated controls	6 days	12 days
Total cells ( × 10^7^)	0.680 ± 0.070	0.505 ± 0.049*	0.651 ± 0.069
Percent AM	98.29 ± 1.22	99.44 ± 0.41	99.42 ± 0.52
Percent PMN	0.30 ± 0.12	0.07 ± 0.12	0.11 ± 0.19
Percent lymphocytes	1.04 ± 0.83	0.34 ± 0.42	0.47 ± 0.41
Percent viable	89.83 ± 3.67	92.79 ± 4.83	92.93 ± 0.51
Protein (mg/mL)	0.11 ± 0.01	0.15 ± 0.04	0.15 ± 0.02
LDH (nmol/min/mL)	69.50 ± 6.37	84.13 ± 25.80	79.66 ± 9.32
β -Glucuronidase (nmol/min/mL)	0.461 ± 0.093	0.497 ± 0.086	0.241 ± 0.048*

Abbreviations: AM, alveolar macrophage; LDH, lactate dehydrogenase; PMN, polymorphonuclear leukocyte. Values are mean ± SD; *n* = 6/group for controls and 3/group for Mn-exposed rats.

* Significantly different from control (*p* < 0.05).
