# Supplementary material for: Translocation of Inhaled Ultrafine Manganese Oxide Particles to the Central Nervous System
Source: Environ Health Perspect. 2006 Apr 20;114(8):1172–8. doi: 10.1289/ehp.9030 (PMC1552007; doi:10.1289/ehp.9030)
Supplement: Supplemental Figures and Tables [file ehp0114-001172s1.pdf]

Supplemental Materials

Characterization of the Oxidation State and Oxide Form of Manganese Oxide UFP

The relative percentage of oxidation states prevalent in a given sample was calculated by normalizing acquired spectra, and minimizing the function Γ(E) with respect to factors a - d, where

$$\Gamma(E) = f_{spectra}(E) - \left(af_{MnO}(E) + bf_{Mn_3O_4}(E) + cf_{Mn_2O_3}(E) + df_{MnO_2}(E)\right)$$

E is electron energy loss and *f* is the normalized Mn L edge intensity for a given Mn oxide after background subtraction. Minimization was carried out using the package, Mathematica® (Wolfram Research, Champaign, IL).

Array Membrane Hybridization

The Atlas™ Rat 1.2 and Rat 1.2 II Array (Clontech) contains a set of nylon membranes with unique probes to 1176 genes for each array. Two array membranes from each treatment group were pre-hybridized using the hybritube15 (Gibco/BRL, Gaithersburg, PA) for 30 min at 68°C with ExpressHyb solution (included in kit) containing heat-denatured sheared salmon testis DNA. The entire label was denatured and added to the mixture and hybridized overnight at 68°C as directed by the protocol. All reagents used were outlined in specific detail in the kit protocol.

Array Analysis

After 3 days of exposure, the membranes were scanned using a phosphorimager (Molecular Imager Fx, BioRad, Hercules, CA), and data collected using AtlasImage™ 1.01 software (Clontech). Data is expressed as fold change differences from the unexposed controls.

TABLE 1S
Rat vs. Human Nasal and Olfactory Parameters [a]

	Rat	Human
Breathing Mode	obligatory nose	nasal/oro-nasal
Area of Nasal Mucosa	~16 cm^2	~105 cm^2
Area of Olfactory Mucosa (% of total mucosa)	~8 cm^2 (50)	5.25 cm^2 (5)
% of Nasal Airflow Going to Olfactory Mucosa	~15	~10
Weight of Olfactory Bulb	~85 ng	~168 ng

[a] Based on Keyhani et al. 1997; Kimbell et al. 1997; Turetsky et al. 2003.

SUPPLEMENTAL FIGURE LEGENDS

Figure 1S. Size distribution of gas phase-generated Mn oxide particles.

Figure 2S. Lung (black) and liver (white) tissue Mn content after inhalation exposure to ultrafine Mn oxide aerosols with both nares patent or with the right naris occluded. The times in the top row indicate the exposure duration; the times in the bottom row indicate post-exposure time. n=4/group. Values are means ± SE; *, p<0.05 *vs*. controls.

FIGURE 1S

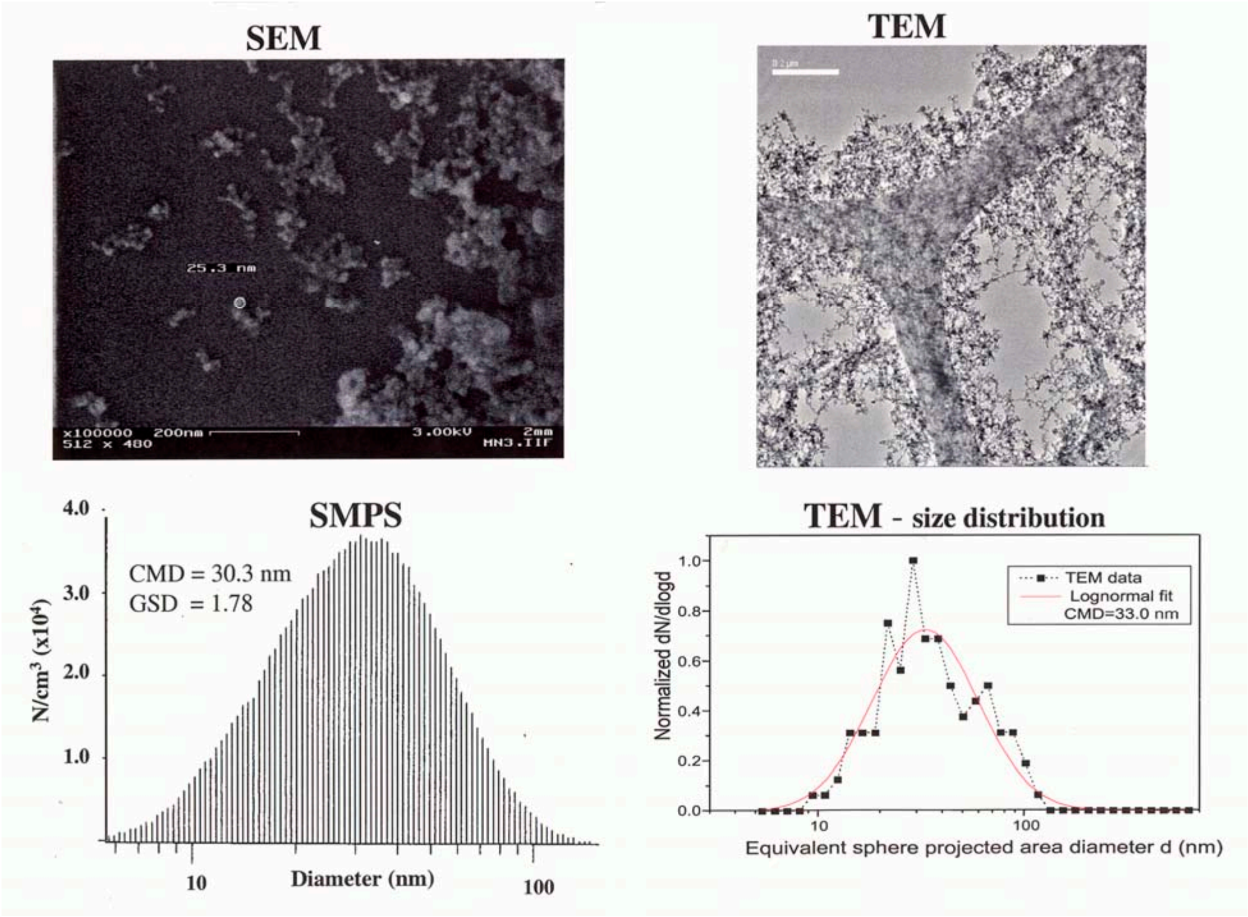
SEM
25.3 nm
x100000 200nm 3.00kV 2mm
512 x 480 MN3.TIF
TEM
SMPS
CMD = 30.3 nm
GSD = 1.78
N/cm³ (x10⁴)
4.0
3.0
2.0
1.0
10
Diameter (nm)
100
TEM - size distribution
TEM data
Lognormal fit
CMD=33.0 nm
Normalized dN/dlogd
1.0
0.8
0.6
0.4
0.2
0.0
10
100
Equivalent sphere projected area diameter d (nm)

FIGURE 2S

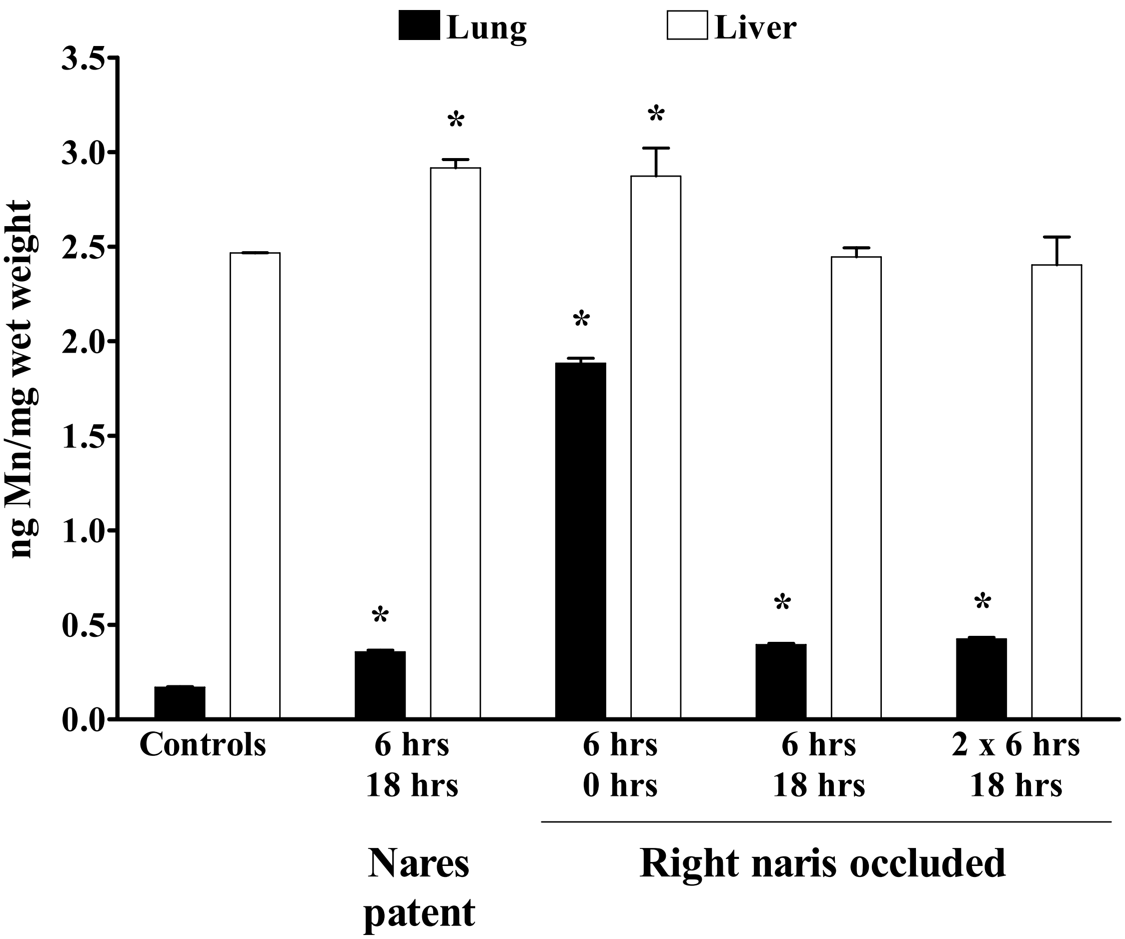

Lung
Liver
ng Mn/mg wet weight
3.5
3.0
2.5
2.0
1.5
1.0
0.5
0.0
*
Controls
6 hrs
18 hrs
6 hrs
0 hrs
6 hrs
18 hrs
2 x 6 hrs
18 hrs
Nares patent
Right naris occluded
